# PRACTICE AND THEORY: THE SIGNIFICANCE OF PARTICIPATING IN CULTURALLY-RELEVANT CRAFTS FOR DIVERSE OLDER ADULTS

**DOI:** 10.1093/geroni/igad104.0139

**Published:** 2023-12-21

**Authors:** Carolyn Adams-Price

## Abstract

In the last 25 years, there has been a great increase in interest in the benefits of creative activities for older adults, especially for dementia patients. More recent research (Adams-Price et al., 2018) has identified specific benefits that healthy older adults get from participating in arts activities. However, little research has featured the benefits of traditional craft activities, especially those prominent in different ethnic groups. The focus of this symposium will be on the benefits of traditional crafts for older adults, especially when those crafts are traditional to one’s culture and have benefits for identity. The proposed symposium will include papers by Chen and Alcantar on the impact of craft participation on diverse older adults. Kaori Otera Chen will be discussing older women weavers in New York State, and the psychological growth they experience from learning and practicing skills associated with weaving, and from creating beautiful pieces. Sandy Alcantar will be discussing a program that she runs for older Hispanic immigrants that promotes participation in traditional crafts to provide income and connect the immigrants to their culture of origin. Adams-Price will be discussing life-span developmental psychology and the sociocultural theory of creativity from Vlad Glaveanu, and their implications of the theory for discussing the significance of long-term participating in culturally-relevant crafts in later life, singly and in groups. She will tie in examples from diverse cultures, including older rural African American women who quilt.

